# COVID-19 free pathways decrease postoperative complications in patients undergoing elective colorectal surgery

**DOI:** 10.1016/j.sipas.2022.100125

**Published:** 2022-09-01

**Authors:** Simona Deidda, Quoc Riccardo Bao, Giulia Capelli, Salvatore Pucciarelli, Luigi Zorcolo, Gaya Spolverato, Angelo Restivo

**Affiliations:** a Department of Surgical Science, University of Cagliari, Cagliari, Italy; b General Surgery 3, Department of Surgical, Oncological and Gastroenterological Sciences, University of Padova, Via Giustiniani 2, Padova 35128, Italy

**Keywords:** COVID-free, Surgical complication, Covid surgery

## Abstract

**Background:**

To reduce the exposition risk to severe acute respiratory syndrome coronavirus 2 (SARS-CoV-2) in surgical patients more prone to develop serious forms of Coronavirus disease 2019, a reorganization that previewed the creation "COVID-19-free" hospitals or units was pursued. The aim of this study was to quantify the effect of clear pathways to reduce the risk of SARS-Cov-2 transmission, on postoperative complications.

**Methods:**

Data of all consecutive patients undergoing surgical procedure for colorectal diseases, between November 2019 and July 2020 in two Italian referral centers, were retrospectively analyzed. Patients were divided into two groups: the ones underwent surgical procedures during the period before the pandemic from November 2019 to March 2020 (Group 1) (before-COVID), and those who underwent surgical procedure from April to July 2020 during the pandemic (Group 2) (during-COVID pandemic).

**Results:**

Overall, 264 patients were collected, 168 (63.4%) in Group 1 and 96 (36.4%) Group 2. Preoperative characteristics were similar between groups; during the pandemic there was a higher proportion of patients who underwent surgical procedures for malignancy compared with the period before the pandemic (92.7% vs 72%; *p* = 0.001). Patients in Group 2 had a lower rate of postoperative general complications (21.9% vs 34.5%; *p* = 0.03) and a lower rate of surgical complications (14.6% vs 25%; *p* = 0.05). No difference in term of medical complications, infections, and intraoperative complications were found. Minimally invasive approach (OR 0.46; 95% CI 0.04–0.83; *p* = 0.01) and isolation of patients (OR, 0.18; 95% CI, 0.04–0.83; *p* = 0.03) were independently associated with lower risk of postoperative complications.

**Conclusion:**

In this cohort study COVID-19-free pathways were significantly associated with low rate of postoperative morbidity in patients undergoing colorectal elective surgery.

## Introduction

The Coronavirus disease 2019 (COVID-19), caused by the severe acute respiratory syndrome coronavirus 2 (SARS-CoV-2), was identified in Wuhan, Hubei province of China in December 2019 [1]. Globally, until March 20th 2021, 122.997.108 cases were confirmed, and a total of 2.715.249 patients have died from this viral infection [2] SARS-CoV-2 mainly affects respiratory tract, despite recent studies showed manifestations of gastrointestinal, central nervous, cardiac, and dermatologic system [3], [4], [5], [6].

In this scenario, hospitals urgently needed to change their organizations, in attempt to free standard and intensive care unit beds for COVID-19 patients. Subsequently, these changes in the health system organization necessitated a sudden mutation in the management of patients with diseases other than COVID-19, with a worrying reduction of elective surgical activities [7], [8], [9].

Given this, a reform of hospitalization system was necessary in order to allow the safe maintenance of elective surgery for patients with life threatening diseases such as oncologic surgery [10], [11], [12]. A reorganization that previewed the creation "COVID-19-free" hospitals or units was pursued to resume elective surgical procedures limiting the exposition risk to SARS-CoV-2 in surgical patients more prone to develop serious forms of COVID-19 [12], [13], [14], [15]. As some studies have shown, these conditions were posed considering that postoperative risk of morbidity and mortality was increased with postoperative diagnosis of SARS-CoV-2 infection especially in centers that were not organized as “COVID-free" [16].

We aimed to identify whether specific protocols to decrease the risk of SARS-Cov-2 transmission resulted in a reduction of postoperative morbidity and mortality, comparing patients tested negative for SARS-CoV-2 and undergoing surgery during the pandemic with those who undergoing the same surgical procedure in a control period.

## Methods

### Study design

All consecutive patients undergoing surgical elective procedure for colorectal diseases, between November 2019 and July 2020, were retrospective selected from a prospectively maintained database of two COVID free high-volume referral centers for Colorectal Surgery. Patients were divided into two groups: the ones underwent surgical procedures from November to March 2020 (Group 1 - before-COVID), and those who underwent surgical procedure from April to July 2020 during the pandemic (Group 2 - during-COVID pandemic). Protective measures and stringent protocols were introduced from April 2020.

In Group 2, we included all patients undergoing elective surgery who were tested negative for SARS-CoV-2 before surgery and did not develop COVID-19 during hospitalization. Patients have been carefully selected and contacted the day before admission to ask if they have experienced in the 15 days before the most common symptoms of COVID-19 infection, such as fever, cough, dyspnea, anosmia, or other respiratory symptoms. Moreover, we investigated if they had strict contact with anyone who presented COVID-19 manifestation or who tested positive for SARS-CoV-2. During the pre-hospitalization period all patients undergoing elective surgery must have had a negative serology test and swab test within 48 h from the day of the scheduled hospitalization. Also, all patients undergoing emergency surgery must have had a negative swab test before the hospitalization.

During the perioperative period the daily ward rounds are limited and performed by a single surgeon, visits were prohibited or limited, and in one of the two centers patients were isolated and hospitalized in a single room. All healthcare staff used maximal individual protective measures, including personal protective equipment (PPE) such as surgical or FFP2 masks, frequent hand sanitation and gloves. Emergency hospitalizations were continued in one of the two institutions even during the pandemic.

Demographics (age, sex) and clinical characteristics including BMI, American Society of Anesthesiology (ASA) status classification, smoking, medications history, diagnoses, surgery details, intraoperative complications, length of stay, and intensive care unit (ICU) admission, were collected.

### Outcomes

The primary outcome was 30-days postoperative complications including surgical and medical complications. The severity of the complications was determined using to the Clavien-Dindo classification [17]. Data concerning post-operative 30-days complications, including both surgical and medical ones, as well as data regarding 30-days postoperative mortality were collected. The following complications were recorded: wound infection, intra-adominal collection, anastomotic leakage, ileus, bleeding, infectious colitis, chest infection, urinary tract infection, renal failure, myocardial infarction, polmonary embolism and cerebrovascular complications. Postoperative surgical complications were classified into superficial, deep, or organ/space SSI.

### Statistical analysis

Statistical analysis was performed using Stata version 13 for Mac (StataCorp, Texas, USA). Continuous variables were expressed as mean [± standard deviation (SD)] or median and interquartile range (IQR); categorical variables as frequencies and percentage. Significant differences between the two groups were tested by χ^2^ test and independent *t*-test for continuous one. The possible relationship between the two groups and postoperative complications was analyzed using a logistic regression model and results are shown as odds ratio (OR) and 95% confidence interval (CI). Univariate and multivariate logistic regression analyses were performed to study the risk of postoperative complications for patients who had undergone surgery.

All tests were two-sided with a level of significance set at *p* < 0.05.

## Results

### Patient's characteristics

Characteristics of patients are summarized in Table 1. Two hundred and sixty-four patients were included in the analysis. One hundred and sixty-eight (63.4%) underwent surgery during the period before the pandemic (Group 1), whereas ninety-six (36.4%) during the pandemic (Group 2). Mean age was 66.5 (±13.9), and 139 (52.6%) of the patients were male. According to the ASA score most patients were ASA II (139 (52.6%)), and the most common diagnoses were malignancy 210 (79.5%). Other diseases recorded included patients undergoing stoma reversal 23 (8.7%), diverticular disease 19 (7.2%) and inflammatory bowel disease (IBD) 12 (4.5%).

**Table 1:** Characteristics of Patients Treated Within pandemic period and before the pandemic.

	Total 264	Group 1 n=168 (63.4%)	Group 2 n=96 (36.4%)	p
**Age, mean (±SD)**	66.5 (±13.9)	66.9 (±13.5)	65.9 (±14.8)	0.55
**Gender, n(%)**				
**Male**	139 (52.6)	93 (55.4)	46 (47.9)	0.35
**Female**	125 (47.3)	75 (44.6)	46 (47.9)	
**BMI, mean (±SD)**	24.4 (±3.9)	24.7 (±4.2)	24 (±3.4)	0.17
**Smoking, n(%)**				
**Yes**	11 (4.2)	5(3.0)	6 (6.2)	0.20
**No**	253 (95.8)	163 (97.0)	90 (93.7)	
**ASA, n(%)**				
**1**	16 (6.1)	13 (7.7)	3 (3.1)	0.40
**2**	139 (52.6)	86 (51.2)	53 (55.2)	
**3**	104 (39.4)	65 (38.7)	39 (40.6)	
**4**	5 (1.9)	4 (2.4)	1 (1.04)	
**Steroids, n(%)**				
**Yes**	5 (1.9)	3 (1.8)	2 (2.1)	0.86
**No**	259 (98.1)	165 (98.2)	94 (97.9)	
**Anticoagulants, n(%)**				0.49
**Yes**	19 (7.2)	12 (6.5)	7 (8.9)	
**No**	245 (92.8)	173 (93.5)	72 (91.1)	
**Antiplatelets, n(%)**				0.52
**Yes**	38 (14.4)	26 (15.5)	12 (12.5)	
**No**	226 (85.6)	142 (84.5)	84 (87.5)	
**Diseases, n(%)**				0.001
**Malignancy**	210 (79.5)	121 (72.0)	89 (92.7)	
**Inflammatory Bowel disease**	12 (4.5)	11 (6.5)	1 (1.04)	
**Diverticular Disease**	19 (7.2)	15 (8.9)	4 (4.2)	
**Stoma reversal**	23 (8.7)	21 (12.5)	2 (2.1)	

Abbreviations: SD, standard deviation; BMI, body mass index; ASA, American Society of Anesthesiology.

The demographics and clinical characteristics for Group 1 and Group 2 were homogeneous. As expected, during the pandemic there was a higher proportion of patients who underwent surgical procedures for malignancy if compared with the period before pandemic (92.7% vs 72.0%; *p* = 0.001) (Table 1).

### Treatment and postoperative complications

**Fig. 1:**
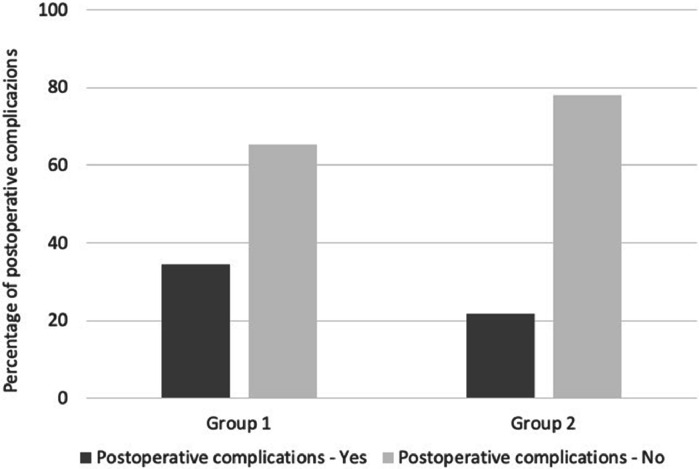
Postoperative complications before (Group 1) and dusrig pandemic (Group 2).

Table 2 showed perioperative characteristics of all patients. Most patients underwent surgery with a laparoscopic approach (n=155, 58.7%), 98 (37.1%) had an open surgery and 11 (4.2%) patients had a robotic approach. Patients in Group 2 had a lower rate of postoperative general complications (21.9% vs 34.5%; *p* = 0.03) (Fig. 1) and a lower rate of surgical complications (14.6% vs 25%; *p* = 0.05). No difference in term of medical complications (6.2% vs 7.7%; *p* = 0.65), infections (18.7% vs 27,4%; *p* = 0.10), intraoperative complications (1% vs 4.2%; *p* = 0.15) were found. Most patients had a complication Clavien-Dindo grade equal to 0 (102, 38.6%) and 1 (102, 38.6%); 27 (10.2%) were graded 2, 27 (10.3%) were graded 3, only 4 (1.5%) were graded 4 and 2 (0.8%) were graded 5, with no difference between the groups. No patients died in Group 2, while the mortality rate in Group 1 was 2.4%. Finally, patients in Group 2 had a shorter length of stay than patients in Group 1 (11.9 ± 11.1 days vs 20.5 ± 21.9 days; *p* < 0.001).

**Table 2:** Operative and postoperative results of patients Treated Within pandemic period and before the pandemic.

	Total 264	Group 1 n=168 (63.4%)	Group 2 n=96 (36.4%)	p
**Operative time, mean (±SD)**	4.2 (±1.7)	4.1 (±1.6)	4.4 (±1.9)	0.12
**Intraoperative complications, n(%)**				0.15
**Yes**	8(3.0)	7 (4.2)	1(1.0)	
**No**	256 (97.0)	161 (95.8)	95 (99.0)	
**ICU admission, n(%)**				0.23
**Yes**	23 (8.7)	12 (7.1)	11 (11.5)	
**No**	241 (91.3)	156 (92.9)	85 (88.5)	
**Surgical approach, n(%)**				0.59
**Open**	98 (37.1)	65 (38.7)	33 (34.4)	
**Robotic**	11 (4.2)	8 (4.8)	3 (3.1)	
**Laparoscopic**	155 (58.7)	95 (56.5)	60 (62.5)	
**Postoperative complications, n(%)**				0.03
**Yes**	79 (29.9)	58 (34.5)	21 (21.9)	
**No**	185 (70.1)	110 (65.5)	75 (78.1)	
**Surgical Complications, n(%)**				0.05
**Yes**	56 (21.2)	42(25.0)	14 (14.6)	
**No**	208 (78.8)	126 (75.0)	82 (85.4)	
**Medical Complications, n(%)**				0.65
**Yes**	19 (7.2)	13 (7.7)	6 (6.2)	
**No**	245 (92.8)	155 (92.3)	90 (93.7)	
**SSI, n(%)**				0.10
**No**	200 (75.8)	122 (72.6)	78 (81.2)	
**Superficial/incisional**	22 (8.3)	14 (8.3)	8 (8.3)	
**Deep/incisional**	9 (3.4)	9 (5.4)	0 (0)	
**Organ/space**	33 (12.5)	23 (13.7)	10 (10.4)	
**Clavien-Dindo Classification, n(%)**				0.45
**0**	102 (38.6)	67 (39.9)	35 (36.5)	
**1**	102 (38.6)	60 (35.7)	42 (43.7)	
**2**	27 (10.2)	17 (10.1)	10 (10.4)	
**3**	27 (10.23)	18 (10.7)	9 (9.4)	
**4**	4 (1.5)	4 (2.4)	0 (0)	
**5**	2 (0.8)	2 (1.4)	0 (0)	
**Hospital Stay (days), median (IQR)**	9(8-64)	9(8-67)	9 (7.5–25)	0.04
**Mortality, n(%)**				0.12
**Yes**	4 (1.5)	4 (2.4)	0 (0)	
**No**	260 (98.4)	164 (97.6)	96 (100)	
**PPE, n(%)**				0.000
**Yes**	83 (31.4)	0 (0)	83 86.46	
**No**	181 (68.6)	168 (100)	13 13.54	
**Hand sanitizer, n(%)**				0.000
**Yes**	83 (31.4)	0 (0)	83 (86.5)	
**No**	181 (68.6)	168 (100)	13 (13.5)	
**Isolation, n(%)**				0.000
**Yes**	34 (12.9)	0 (0)	34 (35.4)	
**No**	230 (87.1)	168 (100)	62 (64.6)	
**Emergency admission, n(%)**				0.001
**Yes**	129 (48.9)	69 (41.1)	60 (62.5)	
**No**	135 (51.1)	99 (58.9)	36 (37.5)	

Abbreviations: ICU, intensive care unit; SSI, Surgical Site Infection; PPE, personal protective equipment.

Data related to the number of emergency admissions were also recorded: during the pandemic period the rate showed to be higher than the previous one (62.5% vs 41.1%; *p* = 0.001)

#### Predictors of postoperative complications

Table 3 reports univariate analysis and shows that patients with postoperative complications had a higher ASA score (ASA 4 (OR 12; 95% CI, 1.02–141.34; *p* = 0.05)), prolonged operative time (OR, 1.20; 95% CI, 1.01–1.37; *p* = 0.03), and patients who had minimally invasive surgery had lower risk of postoperative complications (OR, 0.41; 95% CI, 0.24–0.71; *p* = 0.001). Also, isolation of patients was associated with lower risk of postoperative complications (OR, 0.12; 95% CI, 0.03–0.53; *p* = 0.005), and emergency admission were related with higher risk of postoperative complications (OR, 1.98; 95% CI, 0.16–3.40; *p* = 0.01). The use of Hand sanitizer and PPE led to a lower risk of postoperative complications (both OR, 0.60; 95% CI, 0.32–1.09; *p* = 0.09) The presence of postoperative complications led to a longer length of stay (OR 1.01; 95% CI, 1.00–1.02 *p* = 0.05).

**Table 3:** Univariate analysis for postoperative complications.

	OR	CI (95%)	p
**Age**	1.01	0.99–1.03	0.35
**Gender**			0.36
**Female**	1		
**Male**	1.3	0.75–2.18	
**BMI**	0.99	0.92–1.06	0.81
**ASA**			
**1**	1		
**2**	1.08	0.33–3.58	0.89
**3**	1.45	0.44–4.85	0.54
**4**	12	1.02–141.34	0.05
**Diseases**			
**Malignancy**	1		
**Inflammatory Bowel disease**	1.16	0.34–4.01	0.81
**Diverticular Disease**	1.36	0.51–3.62	0.54
**Stoma reversal**	0.65	0.23–1.82	0.41
**Operative time**	1.20	1.01–1.37	0.03
**Intraoperative complications**			0.76
**No**	1		
**Yes**	0.77	0.15–3.92	
**ICU admission**			0.32
**No**	1		
**Yes**	1.57	0.64–3.79	
**Surgical approach**			
**Open**			
**Minimal invasive approach**	0.41	0.24–0.71	0.001
**Hospital stay**	1.01	1.00–1.02	0.05
**PPE**			0.09
**No**	1		
**Yes**	0.60	0.32–1.09	
**Hand sanitizer**			0.09
**No**	1		
**Yes**	0.60	0.32–1.09	
**Isolation**			0.005
**No**	1		
**Yes**	0.12	0.03–0.53	
**Emergency admission**			0.01
**No**	1		
**Yes**	1.98	1.16–3.40	

Abbreviations: BMI, body mass index; ASA, American Society of Anesthesiology; ICU, intensive care unit; SSI, Surgical Site Infection; PPE, personal protective equipment.

Multivariate analysis is reported in Table 4 and showed that minimally invasive approach (OR 0.46; 95% CI 0.04–0.83; *p* = 0.01) and isolation of patients (OR, 0.18; 95% CI, 0.04 - 0.83; *p* = 0.03) were independently associated with lower risk of postoperative complications.

**Table 4:** Multivariate analysis for postoperative complications.

	OR	CI (95%)	p
**Operative time**			0.13
**No**	1		
**Yes**	1.14	0.96–1.35	
**Surgical approach**			0.01
**Open**	1		
**Minimal invasive approach**	0.46	0.04–0.83	
**Isolation**			0.03
**No**	1		
**Yes**	0.18	0.04–0.83	
**Emergency admission**			0.72
**No**	1		
**Yes**	1.12	0.60–2.10	

## Discussion

This retrospective study compares postoperative morbidity rates between patients undergoing abdominal surgical procedures during the pandemic period, with those undergone the same surgical treatments in a control period. The aim was to quantify the effect of clear pathways to reduce the risk of SARS-Cov-2 transmission, on postoperative complications.

Our results demonstrated that strict compliance on protective measures and stringent protocols allows to safely carry out elective surgical activity during pandemic without compromising short-term postoperative outcomes. Moreover, these results showed that isolation and hospitalization of the patients in a single room significantly reduce the risk of postoperative complications.

Recently some studies have analyzed short-term postoperative outcomes in surgical patients during the pandemic and showed that complications were more common during this period. However, the primary aim of these studies was to examine early surgical morbidity and mortality in patients with COVID-19 compared with patients without the disease [16,[18], [19], [20], [21], [22]]. Postoperative outcomes in SARS-CoV-2-infected patients are worse than pre-pandemic: 30-days mortality was close to 20–25% [18,23], and pulmonary, thrombotic and surgical postoperative complications dramatically increased [20].

No study has specifically analyzed the impact of anti-COVID-19 measures on elective surgical activity and postoperative outcomes in patients who did not develop the infection. A recent study compared patients undergoing elective surgery during the pandemic in a COVID- 19 – free surgical pathways with patients undergoing surgery in a no defined pathways, to determine whether COVID-19–free surgical pathways were associated with lower postoperative pulmonary complication rates [15]. Data from 9171 patients showed that complications and death after surgery were lower for patients treated in COVID-free units. Pulmonary complications for those in COVID-19-free units were 2.2% compared to 4.9%, the rates of contracting COVID-19 around the time of surgery were 2.1% versus 3.6%, and the rates of death was also lower (0.7% vs 1.7%).

Otherwise, we focused our analysis on patients who did not develop SARS-Cov-2 infection in the perioperative period and up to 30-day after surgery. Interestingly, we identified that, as well as the intraoperative period [24], [25], [26], also the postoperative course should be implemented with some interventions in order to reduce postoperative morbidity. We specifically identified that isolation, hospitalization in a single room and visits prohibited (or limited), led to a control of postoperative complications. Also, a minimally invasive approach showed a very positive effects and advantages on postoperative complications, as already showed by previous studies [27,28]. This retrospective study documents that postoperative morbidity after colorectal elective surgery was significantly lower during the pandemic, whether COVID-19-free pathways were followed. With an implementation of intra-hospital protocols during the pandemic, we find that elective surgery could be performed safely for both patients and caregivers. Moreover, these hospitalization protocols should be validated and used in daily clinical practice.

There were many acknowledged limitations. The main bias is related to the retrospective design. As with every retrospective series, bias related to patient selection and heterogeneity of clinical practice among the two involved centers. The potential biases are largely compensated by the strength of numbers and by the fact that data were obtained from prospectively maintained datasets from two referral colorectal cancer centers.

## Declaration of Competing Interest

All authors declare that there was no conflict of interest.

## References

[1] Guo Y.R., Cao Q.D., Hong Z.S., Tan Y.Y., Chen S.D., Jin H.J. (2020). The origin, transmission and clinical therapies on coronavirus disease 2019 (COVID-19) outbreak - an update on the status. Mil Med Res.

[2] Center for Disease Control and Prevention, COVID data Tracker, Available from: https://covid.cdc.gov/covid-data-tracker/#datatracker-home, Accessed on March 2021.

[3] Alam S.B., Willows S., Kulka M., Sandhu J.K. (2020). Severe acute respiratory syndrome coronavirus 2 May be an underappreciated pathogen of the central nervous system. Eur J Neurol.

[4] Deidda S., Tora L., Firinu D., Del Giacco S., Campagna M., Meloni F. (2021). Gastrointestinal coronavirus disease 2019: epidemiology, clinical features, pathogenesis, prevention, and management. Expert Rev Gastroenterol Hepatol.

[5] Gottlieb M., Long B. (2020). Dermatologic manifestations and complications of COVID-19. Am J Emerg Med.

[6] Larson A.S., Savastano L., Kadirvel R., Kallmes D.F., Hassan A.E., Brinjikji W. (2020). Coronavirus disease 2019 and the cerebrovascular-cardiovascular systems: what do we know so far?. J Am Heart Assoc.

[7] Zarrintan S. (2020). Surgical operations during the COVID-19 outbreak: should elective surgeries be suspended?. Int J Surg.

[8] COVIDSurg Collaborative (2020). Elective surgery cancellations due to the COVID-19 pandemic: global predictive modelling to inform surgical recovery plans. Br J Surg.

[9] Francis N., Dort J., Cho E., Feldman L., Keller D., Lim R. (2020). SAGES and EAES recommendations for minimally invasive surgery during COVID-19 pandemic. Surg Endosc.

[10] Liang W., Guan W., Chen R., Wang W., Li J., Xu K. (2020). Cancer patients in SARS-CoV-2 infection: a nationwide analysis in China. Lancet Oncol.

[11] Philouze P., Cortet M., Quattrone D., Céruse P., Aubrun F., Dubernard G. (2020). Surgical activity during the COVID-19 pandemic: results for 112 patients in a French tertiary care center, a quality improvement study. Int J Surg.

[12] Spolverato G., Capelli G., Restivo A., Bao Q.R., Pucciarelli S., Pawlik T.M. (2020). The management of surgical patients during the coronavirus disease 2019 (COVID-19) pandemic. Surgery.

[13] Restivo A., De Luca R., Spolverato G., Delrio P., Lorenzon L., D'Ugo D. (2020). The need of COVID19 free hospitals to maintain cancer care. Eur J Surg Oncol.

[14] Cavaliere D., Parini D., Marano L., Cipriani F., Di Marzo F., Macrì A. (2021). Surgical management of oncologic patient during and after the COVID-19 outbreak: practical recommendations from the Italian society of surgical oncology. Updates Surg.

[15] Glasbey J.C., Nepogodiev D., Simoes J.F.F., Omar O., Li E., Venn M.L. (2020). Elective cancer surgery in COVID-19–free surgical pathways during the SARS-CoV-2 pandemic: an international, multicenter, comparative cohort study. J Clin Oncol.

[16] Abate S.M., Mantefardo B., Basu B. (2020). Postoperative mortality among surgical patients with COVID-19: a systematic review and meta-analysis. Patient Saf Surg.

[17] Dindo D., Demartines N., Clavien P.A. (2004). Classification of surgical complications. Ann Surg.

[18] COVIDSurg Collaborative (2020). Mortality and pulmonary complications in patients undergoing surgery with perioperative SARS-CoV-2 infection: an international cohort study. Lancet.

[19] Lei S., Jiang F., Su W., Chen C., Chen J., Mei W. (2020). Clinical characteristics and outcomes of patients undergoing surgeries during the incubation period of COVID-19 infection. EClinicalMedicine.

[20] Doglietto F., Vezzoli M., Gheza F., Lussardi G.L., Domenicucci M., Vecchiarelli L. (2020). Factors associated with surgical mortality and complications among patients with and without coronavirus disease 2019 (COVID-19) in Italy. JAMA Surg.

[21] Jonker P.K.C., van der Plas W.Y., Steinkamp P.J., Poelstra R., Emous M., van der Meij W. (2021). Perioperative SARS-CoV-2 infections increase mortality, pulmonary complications, and thromboembolic events: a Dutch, multicenter, matched-cohort clinical study. Surgery.

[22] Cano-Valderrama O., Morales X., Ferrigni C.J., Martín-Antona E., Turrado V., García A. (2020). Acute care surgery during the COVID-19 pandemic in Spain: changes in volume, causes and complications. A multicentre retrospective cohort study. Int J Surg.

[23] Gammeri E., Cillo G.M., Sunthareswaran R., Magro T. (2020). Is a “COVID-19-free” hospital the answer to resuming elective surgery during the current pandemic? Results from the first available prospective study. Surgery.

[24] Badia J.M., Rubio-Pérez I., López-Menéndez J., Diez C., Al-Raies Bolaños B., Ocaña-Guaita J. (2020). The persistent breach between evidence and practice in the prevention of surgical site infection. Qualitative study. Int J Surg.

[25] Wick E.C., Hobson D.B., Bennett J.L., Demski R., Maragakis L., Gearhart S.L. (2012). Implementation of a surgical comprehensive unit-based safety program to reduce surgical site infections. J Am Coll Surg.

[26] Dieplinger B., Egger M., Jezek C., Heinisch-Finke C., Altendorfer C., Pernerstorfer T. (2020). Implementation of a comprehensive unit-based safety program to reduce surgical site infections in cesarean delivery. Am J Infect Control.

[27] van der Pas M.H., Haglind E., Cuesta M.A., Fürst A., Lacy A.M., Hop W.C. (2013). Laparoscopic versus open surgery for rectal cancer (COLOR II): short-term outcomes of a randomised, phase 3 trial. Lancet Oncol.

[28] Prete F.P., Pezzolla A., Prete F., Testini M., Marzaioli R., Patriti A. (2018). Robotic versus laparoscopic minimally invasive surgery for rectal cancer: a systematic review and meta-analysis of randomized controlled trials. Ann Surg.

